# ASPSCR-1 and Sirt-5 alleviate Clonorchis liver fluke r*Cs*NOSIP-induced oxidative stress, proliferation, and migration in cholangiocarcinoma cells

**DOI:** 10.1371/journal.pntd.0011727

**Published:** 2023-11-10

**Authors:** Meng Bian, Shan Li, Hanzong Zhou, Lijun Bi, Yong Shen, Chen Tingjin, Xinbing Yu, Yan Huang, Qingxia Xu

**Affiliations:** 1 Department of Clinical laboratory, Affiliated Cancer Hospital of Zhengzhou University & Henan Cancer Hospital, Zhengzhou, Henan, People’s Republic of China; 2 Zhengzhou Key Laboratory of Digestive System Tumor Marker Diagnosis, Zhengzhou, Henan, People’s Republic of China; 3 Department of Pathology and Pathophysiology, School of Medicine, Henan University of Chinese Medicine, Zhengzhou, Henan, People’s Republic of China; 4 The fifth Clinical Medical College of Henan University of Chinese Medicine (Zhengzhou People’s Hospital), Zhengzhou, Henan, People’s Republic of China; 5 Institute of Biophysics, Chinese Academy of Scineces, Beijing, People’s Republic of China; 6 Key Laboratory for Tropical Diseases Control, Ministry of Education, Sun Yat-sen University, Guangzhou, People’s Republic of China; 7 Department of Pathology, Duke University School of Medicine, Durham, North Carolina, United States of America; University of Liverpool, UNITED KINGDOM

## Abstract

**Background:**

Clonorchiasis, caused by the infection of *Clonorchis sinensis* (*C*. *sinensis*), is a kind of neglected tropical disease, but it is highly related to cholangiocarcinoma. It has been well known that NO from chronic inflammation responses are thought to be a major component of the damage and ultimate carcinogenesis ESPs such as nitric oxide synthase interacting protein (NOSIP) are thought to enhance the damage. The objective of this study was to identify the protein candidates interact with recombinant *Cs*NOSIP (r*Cs*NOSIP) and explore their role involved in CCA development or progression.

**Methods:**

We applied HuProt microarray containing 21,000 probe sets for a systematic identification of r*Cs*NOSIP-binding proteins and grouped binding hits by gene function. Pull-down assays were used to confirm the interaction of r*Cs*NOSIP with alveolar soft part sarcoma (ASPSCR-1) and sirtuins 5 (Sirt-5). ASPSCR-1/Sirt-5 over-expression and siRNA knockdown experiments were employed for obtain of ASPSCR-1/Sirt-5 high or low expression (ASP-oe/Sirt5-oe or ASP-si/Sirt5-si) cholangiocarcinoma cell line (CCLP-1) cells. Nitric oxide (NO) and reactive oxygen species assay (ROS) as well as cell proliferation and wound-healing assays were performed to observe the effect of r*Cs*NOSIP on ASP-oe/Sirt5-oe or ASP-si/Sirt5-si CCLP-1 cells.

**Results:**

Seventy candidate proteins protein "hits" were detected as r*Cs*NOSIP-binding proteins by HuProt microarray and bioinformatics analysis. Pull down assay showed that ASPSCR-1 and Sirt-5 could interact with r*Cs*NOSIP. In addition, endotoxin-free-r*Cs*NOSIP could increase the production of NO and ROS and promote the migration of CCLP-1 cells, while its effect on enhancing cell proliferation was not significant. Furthermore, ROS/NO production, proliferation, or migration were increased in ASP-si or Sirt5-si CCLP-1 cells but decreased in Asp-oe or Sirt5-oe CCLP-1 cells when stimulated with r*Cs*NOSIP.

**Conclusions:**

Our findings suggest that *Cs*NOSIP as a component of *Cs*ESPs might promote the development and invasion of CCA and Sirt5/ ASPSCR1 as host molecules might play a novel protective role against adverse stimulus during *C*. *sinensis* infection. This work supports the idea that *Cs*ESPs induce the occurrence and progression of CCA through ROS/RNS-induced oxidative and nitrative DNA damage.

## Author summary

*Clonorchis sinensis* (*C*. *sinensis*) is prevalent in China, Korea, and Vietnam, and 15–20 million people are estimated to be infected by this fluke, creating a socio-economic burden in epidemic regions. It is generally believed that excretory-secretory products from *C*. *sinensis* (*Cs*ESPs) can directly interact with the biliary epithelium and induce host inflammatory response. Our previous studies have manifested that *Cs*NOSIP as a component of *Cs*ESPs can trigger nitrative and oxidative stress by enhancing the release of NO and reactive oxygen species (ROS) in macrophage. In the current study, we identified r*Cs*NOSIP-binding proteins by HuProt microarray. In addition, we demonstrated that ASPSCR-1 and Sirt-5 could interact with r*Cs*NOSIP by pull down assay. Moreover, r*Cs*NOSIP could increase the production of NO and ROS and promote the migration of human CCLP-1 cells by using NO assay, detection of ROS assay as well as wound-healing assays. Our study provided new information about r*Cs*NOSIP in promoting CCA invasion and metastasis as well as a novel protective role of SIRT5/ ASPSCR1 against adverse stimulus and support a basis for further discovery of drug targets for prevention and control of clonorchiasis.

## Introduction

*Clonorchis sinensis* (*C*. *sinensis*), the causative agents of clonorchiasis, is an important food-borne parasite endemic throughout Southeast Asia [1,2,3]. Clonorchiasis is prevalent in China, Korea, and Vietnam, and 15–20 million people are estimated to be infected by this fluke, creating a socio-economic burden in epidemic regions [4]. Because of the high correlation between clonorchiasis and cholangiocarcinoma (CCA), *C*. *sinensis* was reclassified as a group -I biocarcinogen for CCA by the International Agency for Research on Cancer (IARC) in 2009 [4–7]. This biocarcinogen has been included in control programs of neglected 57 tropical diseases by WHO [5]. It is generally believed that excretory-secretory products from *C*. *sinensis* (*Cs*ESPs) can directly interact with the biliary epithelium and induce host inflammatory response [8,9]. During chronic inflammation, excessive nitrogen and oxygen radicals especially nitric oxide (NO) generated by inflammatory and epithelial cells may exert cytotoxic and mutagenic effects through inducing nitrative and oxidative DNA damage [10,11]. NO is accordingly considered as a carcinogen that promotes the occurrence and progression of CCA via chronic inflammation [12]. Certain ESPs, such as NOSIP, are thought to cause further damage through excess NO production. Therefore, understanding the exact function of key molecules in *Cs*ESPs that are related to NO production or its reactive intermediates will facilitate illumination of CCA pathogenesis during *C*. *sinensis* infection.

NO is produced from a reaction catalyzed by three major isoforms of nitric oxide synthase (NOS) including neuronal (nNOS/NOS1), inducible (iNOS/NOS2), and endothelial (eNOS/NOS3) [13,14]. As an eNOS interaction protein, NO synthase interacting protein (NOSIP) possibly affects eNOS activity and regulates NO production [15,16]. Our previous studies have manifested that *C*. *sinensis* NOSIP (*Cs*NOSIP) as a component of *Cs*ESPs can trigger nitrative and oxidative stress by enhancing the release of NO and reactive oxygen species (ROS) in macrophage. However, *Cs*NOSIP-mediated molecular mechanism in CCA pathologic process remains as yet unclear. In the present study, HuProt microarray was employed for exploring protein binding partners to recombinant *Cs*NOSIP protein (r*Cs*NOSIP). Potential positive proteins interaction with r*Cs*NOSIP was screened through bioinformatics analysis. The interaction of r*Cs*NOSIP with ASPSCR-1 and Sirt-5, two screened proteins closely related to tumor progression, was confirmed by GST-pull down assay. The influence of r*Cs*NOSIP on proliferation, migration, and NO/ROS production in CCLP1 cells line when over expression and low expression of ASPSCR-1 and Sirt-5 was investigated. Our study will provide new information about parasite-host interaction in CCA pathogenesis and support a basis for further discovery of drug targets for prevention and control of clonorchiasis.

## Materials and methods

### Protein expression and purification

Recombinant *Cs*NOSIP protein (r*Cs*NOSIP) was produced as previous method with slight modification [17]. Briefly, pET-28a-(+)-*Cs*NOSIP was constructed and transformed into *E*. *coli* (DE3) (Promega). Induced by 0.2 mM isopropyl-β-D-thiogalactopyranoside (IPTG), the protein was expressed at 30°C for 12 h in Luria-Bertani medium and purified by Ni-nitrilotriacetic acid (NTA) chromatography. The purified r*Cs*NOSIP was analyzed by 12% SDS-PAGE stained with Coomassie brilliant blue G-250 and the concentration of recombinant soluble protein concentration was detected by the BCA protein assay kit (Novagen, USA). The protein was treated by AffinityPa Detoxi-Ge Endotoxin Removing Gel (Thermo, USA) and Limulus test was used to make sure that the endotoxin had been removed. Then, the concentration of r*Cs*NOSIP was determined by BCA protein after endotoxin cleanup.

### Detection of target protein by HuProt microarray assay

The HuProt microarray assay procedures were described previously [18]. Briefly, microarrays were incubated with blocking-buffer (3% BSA in 1× PBS, pH 7.4) for 3 h, shaking at 4°C. Purified *Cs*NOSIP was labeled with Cy3 (GE Healthcare, Little Chalfont, UK) and diluted with binding-buffer (1% BSA in 1×PBS, pH 7.4). The diluted *Cs*NOSIP-Cy3 (15 μg) incubation was performed at 4°C overnight. After washing 3 times with PBST, then twice with ultra-pure water, array was scanned in a LuxScan 10K-A (CapitalBio Corporation, Beijing, China). The scanned fluorescence data was analyzed with GenePix Pro 6.0 software (Molecular Devices). To generate candidate lists, median normalization was performed for each microarray, and the cutoff value was defined as mean+2*SD (95%). Then, the normalized SNRs for *Cs*NOSIP and BSA were set as SNR (+) and SNR (−), and Fold change was defined as SNR (+)/SNR (−). The candidate targets were defined as proteins with SNR (+) ≥5.35 (95%CI) and Fold change ≥1.2 [19].

### Bioinformatics analysis

A total of 70 candidate targets were listed for subsequent bioinformatics analysis. GO and the KEGG were used for protein functional annotation. Functional enrichment on the proteins was achieved by the R package cluster Profiler (v4.0.5). KEGG pathway and GO terms with *P* values < 0.05, were considered significantly enriched based on the hyper geometric distribution.

### Cell culture

Human cholangiocarcinoma cell line (CCLP-1) was gifted from Professor Bi’s lab at the Institute of Biophysics, Chinese Academy of Sciences.CCLP-1 cell lines was cultured in DMEM including 1% penicillin, 1% streptomycin and 10% FBS at 37°C under 5% CO_2_.

### Measurement of mRNA expression by quantitative real-time PCR

The extraction of total RNA, complementary DNA synthesis and PCR amplification were carried out as described previously [20]. Briefly, total RNAs from control group (CCLP1 cells without *Cs*NOSIP) and *Cs*NOSIP+ group (CCLP1 cells with 5 μg/ml *Cs*NOSIP for 24 h) were extracted in Trizol reagent (Invitrogen) according to the manufacturer’s instructions. RNA concentrations were detected by nucleic acid/protein analyzer (Beckman Coulter, USA). The preparation of the first-strand cDNA was carried out using RT-PCR Kit (TIANGEN, Beijing, China) with the same quantity of total RNA as the template (1 μg).

Quantitative real-time PCR (qRT-PCR) was employed to study the relative expression of ASPSCR1 and Sirt 5 in CCLP1 induced by *Cs*NOSIP. All PCR was carried out using a Bio-Rad CFX96 real-time PCR system (Bio-Rad Laboratories). The forward and reverse primers for ASPSCR1 were 5’-TGACGCGCCACTCCAAAAACT-3’ and 5’-CCA AATACTCTAAGACCGCCGC-3’, primers for Sirt 5 were 5’-CCCAGAACATCGATGAGC-3’ and 5’-GCCACAACTCCACAAGAGG-3’, respectively [21]. The housekeeping β-actin gene used as a reference for mRNA quantification. The forward and reverse primers for β-actin were 5’- CTTCCTTCCTGGGCATGG-3’ and 5’-GCCGCCAGACAGCACTGT-3’. Quantitative RT-PCR reactions based on SYBR-Green I fluorescence (TIANGEN, Beijing, China) were performed by using Bio-Rad iQ5 instrument (Bio-Rad, USA). The PCR amplification program was 95°C for 30 s, 40 cycles of 95°C for 5 s, and 60°C for 20 s. The iQ5 software was used to analyze the relative quantification according to the 2^−ΔΔCt^ method [22].

### Pull-down assays

To investigate the interaction of r*Cs*NOSIP with ASPSCR-1 and Sirt-5, GST tagged r*Cs*NOSIP was prepared for the GST-pull down assay. Expression vectors pET-28a-Flag-ASPSCR-1, pET-28a-Flag-Sirt 5 and pET-28a-His-GST-*Cs*NOSIP were generated by inserting the corresponding tag fusion fragment into pET-28a. Gene fragment synthesis and vector construction were performed by GenScript Biotech Corporation. His-GST-*Cs*NOSIP was purified as described above. 50 μg purified GST-r*Cs*NOSIP was immobilized by GST affinity beads (Roche Applied Science, Indianapolis, IN) and incubated with cells lysate containing ASPSCR-1(or Sirt 5). After shaking at room temperature for 2 h, the beads were pelleted and washed twice with PBS buffer. The bound protein was eluted with 10 mM GSH buffer then resolved by SDS-PAGE and detected by rabbit anti-GST- antibody or mouse anti-Flag. Cell lysate without ASPSCR-1(or Sirt 5) and purified GST protein were used as controls for nonspecific interactions between GST-r*Cs*NOSIP and ASPSCR-1(or Sirt 5).

### ASPSCR-1/Sirt 5 over-expression and siRNA knockdown experiments

To investigate whether ASPSCR1 and Sirt 5 protein were involved in the stress response in CCLP 1 cell lines with 5 μg /mL purified *Cs*NOSIP, cell lines of different expression levels of ASPSCR1 and Sirt 5 were generated. Plasmids (pcDNA3.1-ASPSCR-1 and pcDNA3.1-Sirt 5) were from GenScript Biotech Corporation. CCLP-1 cells were transfected with above plasmids using Polyjet reagent (SignaGen) according to the manufacturer’s protocol. After 48 or 72 h, the cells were collected and lysed in lysis buffer, then detected by western blot with anti-ASPSCR1 mouse antibody or anti-Sirt 5 mouse antibody. Alternatively, cells were stimulated by *Cs*NOSIP. For low expression of ASPSCR-1/Sirt 5 in CCLP-1 cell lines, knockdown experiments were performed using gene-specifific siRNAs (siRNA 1 group and siRNA 2 group). siRNAs were chemically synthesised by RIBOBIO (RIBOBIO, Guangzhou, China). Sequences were as follows: ASPSCR1 siRNA 1, 5′-CAAUGCCAAGCUGGAGAUGTT-3′; ASPSCR1 siRNA 2, 5′-CAUCUCCAGCUUGGAUUGTT-3′; Sirt 5 siRNA 1,5′-GGAGAUCCAUGGUAGCUUATT-3′; Sirt 5 siRNA 2,5′- UAAGCUACCAUGGAUCUCCTT-3′. siRNA transfections were performed using Lipofectamine RNAiMax transfection reagent (Life Technologies), according to the manufacturer’s specifications. Whole cell lysate was prepared at 72 h after transfection for western blot analysis. Alternatively, cells were collected for quantitative real-time PCR(qRT-PCR)analysis.

### Western blot

Western blot was carried out as described by Xu et al [3]. Briefly, Cells were lysed in RIPA buffer and then loaded onto 12% SDS-polyacrylamide gels for electrophoresis. Proteins on gels were transferred onto a nitrocellulose membrane, and was assayed by Ponceau S staining. Stained membrane was washed with TBST solution (20 mM Tris-HCl, 0.15 M NaCl, 0.05% Tween-20, pH 7.5) immediately and blocked in 5% (w/v) non-fat milk in TBST. The membrane was incubated with corresponding primary antibody (anti-GST- antibody from CoWin Biotech, anti-Flag from YEASEN, anti-ASPSCR1 antibody from Sino Biological, anti-Sirt 5 from Proteintech, anti-β-actin from abcam) at room temperature for 2 h. After 3 times of TBST washing, membrane was incubated with a secondary horseradish peroxidase linked anti-rabbit IgG or anti mouse IgG (1:10000, GE Healthcare). Chemiluminescent substrate (enhanced chemiluminescence -plus substrate, GE Healthcare) was used for detection according to the manufacturer’s instructions.

### Quantitative real-time PCR (qRT-PCR)

RNA was extracted from harvest cells using a RNA preppure Kit (TIANGEN, Beijing, China) according to the manufacturer’s instructions. The first-strand cDNA was synthesized from the total RNA using RNA First-Strand cDNA Kit (TIANGEN, Beijing, China). Subsequently, qRT-PCR was conducted using gene-specific primers to determine the expression levels of mRNAs. Following initial denaturation at 95°C for 30 s, 40 cycles of PCR amplification were performed at 95°Cfor 5 s and 60°C for 30 s, with a dissociation curve at the end of the amplification reaction. The qRT-PCR data were calculated by the 2^-△△CT^ method [4] while the expression level of β- actin was used for normalization. The results of qRT-PCR were analyzed using GraphPad Prism (version 7.0). Differences between groups were statistically analyzed using analysis of variance (ANOVA). A *P* value < 0.05 was considered statistically significant.

### NO and ROS measurement

CCLP-1 cells were transfected with pcDNA3.1-ASPSCR-1 or pcDNA3.1-Sirt 5 for 48 h and then stimulated by adding 5 μg/mL purified *Cs*NOSIP for 24 h. Cultured cells and medium were collected separately. NO concentrations were quantified using a nitric oxide assay kit (Beyotime Biotechnology, Shanghai, China). Briefly, cell culture supernatants and standards of NaNO_2_ (0,1, 2, 5, 10, 20, 40, 60, 100 μM) were added into individual wells of a 96-well plate. Fifty microliters of Griess reagent I and Griess reagent II were then dripped into each well successively. After 3 min incubation, the absorbance was measured at 540 nm utilizing a microplate reader, and the concentration of NO was determined.

Reactive Oxygen Species Assay Kit (2,7-Dichlorodi-hydrofluorescein diacetate, DCFH-DA; Applygen Technologies, Beijing, China) were used to measure the quantity of reactive oxygen species (ROS) in the cholangiocarcinoma cell (CCLP-1). The dye loading was performed by incubating the cells with 10 μM DCFH-DA at 37°C for 60 min. The production of ROS was examined using a Flow cytometer (BD FACS Canto, BD Biosciences USA) by measuring the fluorescence intensity of DCF at an excitation wavelength of 488 nm and an emission wavelength of 525 nm.

### Cell proliferation and Wound-healing assays

The proliferation activity of cells was detected by CCK-8 method. Cells were transfected for 48 h, then were seeded in 96-well plates at a density of 5000 cells per well. After cells attachment, the fresh medium with or without 5 μg/mL purified *Cs*NOSIP protein was added into cells, incubating for 24 h. The proliferation activity of cells was detected by Cell Counting Kit-8 (Beyotime, China), according manufacturer’s instructions. In briefly, the medium was removed and replaced with fresh medium containing 10% CCK-8 solution. Cells were incubated for 3 h at 37°C. Then the 450 nm wave-length absorption values were measured using a microplate reader (Multiskan FC, Thermo Scientific, USA). The experiment was performed three times with five replicates for each sample.

Cell migration was analyzed by the wound healing assay [23]. In brief, cells were transfected for 48 h and several wound lines were scratched vertically to the bottom with a 200 ml pipette tip. After being washed with PBS three times, cells were incubated in growth medium containing *5* μg/mL *Cs*NOSIP protein without serum. The wound areas were determined at 24 h with microscope (Nikon, Japan). According to the collected image data, the data analysis was performed by ImageJ (v1.8.0). The calculation formula of the result was descripted by the calculation formula percentage of cell migration = (initial scratch area- final scratch area) /initial scratch area × 100%.

### Statistical analysis

Statistical analysis was performed using GraphPad Prism software. All data were presented as mean ± SD. For comparison of groups, one-way ANOVA and Student’s t test were used, and one-way ANOVA using SPSS software for windows (version 17.0; SPSS, Inc., IL). All graphs were performed using GraphPad Prism software. *P* < 0.05 was taken to be significant.

## Results

### Expression, endotoxin removal and purification of r*Cs*NOSIP

**Fig 1:**
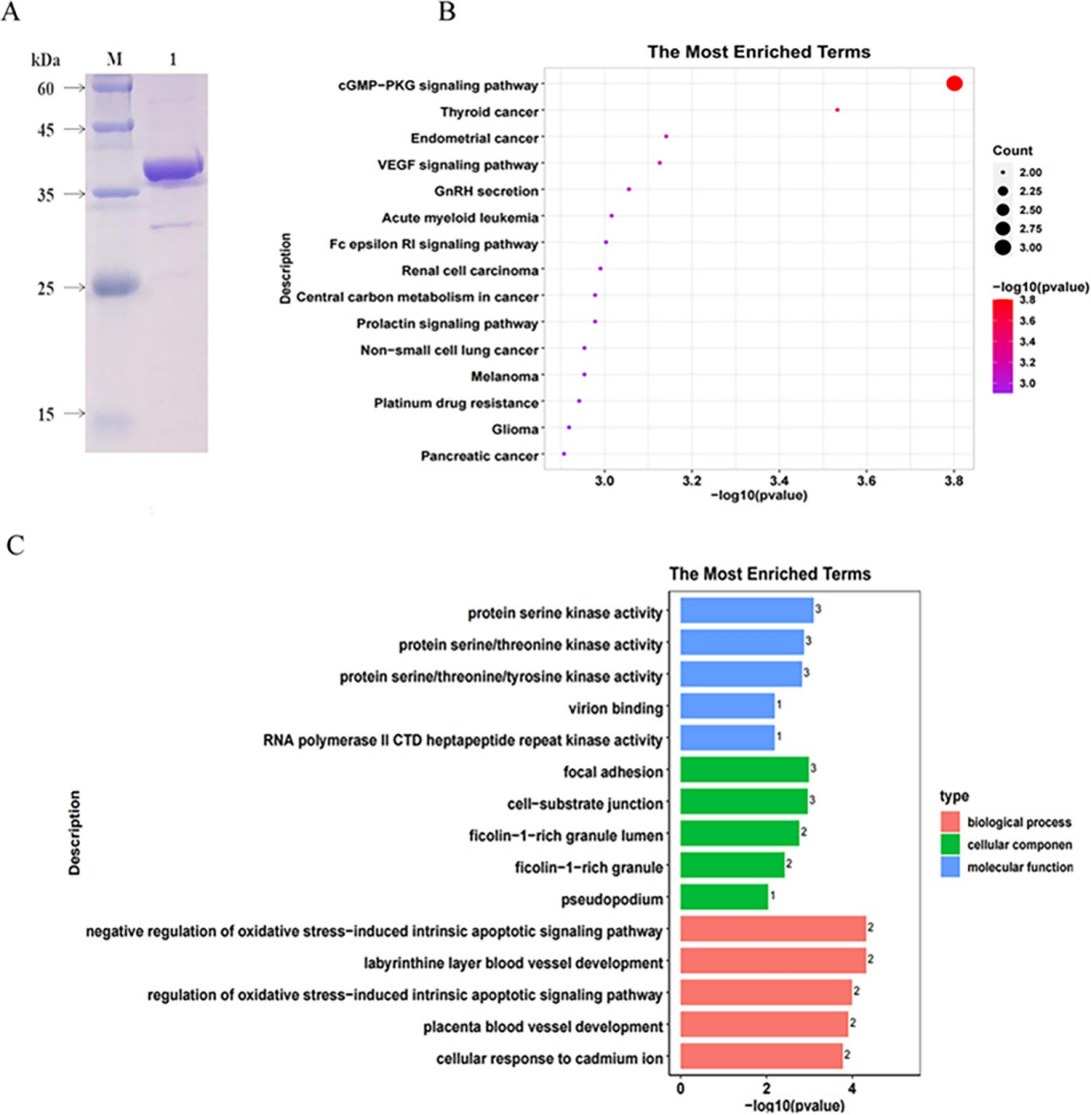
SDS-PAGE of purified *Cs*NOSIP and functional enrichment of biological processes of proteins identified by protein array. (A) SDS-PAGE of purified protein. Lane M, protein marker; lane 1, purified *Cs*NOSIP on Coomassie Brilliant Blue (CBB) stained SDS-PAGE gel. (B) Enrichment of biological processes in terms of gene ontology (GO) categories. (C) Enrichment of Kyoto Encyclopedia of Genes and Genomes (KEGG) categories.

Recombinant *Cs*NOSIP was expressed in *E*. *coli* (DE3) and purified using Ni^2+^-NTA chromatogr-aphy (S1 Fig). The purified r*Cs*NOSIP of approximately 36 kDa were identified by SDS-PAGE (Fig 1A). The concentration of the recombinant His tag-*Cs*NOSIP fusion protein was about 1.25 mg/ml, while the concentration of endotoxin-free r*Cs*NOSIP was about 645 μg/ml. We applied a HuProt human proteome microarray containing over 21,000 human proteins for a systematic identification of *Cs*NOSIP-binding. Seventy candidate proteins were positived as r*Cs*NOSIP-binding proteins by HuProt microarray and bioinformatics analysis (S1 Table).

### Functional enrichment analysis

Go enrichment indicated that these candidate proteins were associated with biological process including protein serine/threonine/tyrosine kinase activity, and regulation of oxidative stress-induced intrinsic apoptotic signaling pathway (Fig 1C). In KEGG pathway, we found that the candidate proteins were associated with various tumors progression, such as thyroid cancer, endometrial cancer, especially highly correlated with cGMP-PKG (Fig 1B).

### Transcriptional levels of ASPSCR1 and Sirt 5 in CCLP1 cells stimulated with *Cs*NOSIP by QPCR

**Fig 2:**
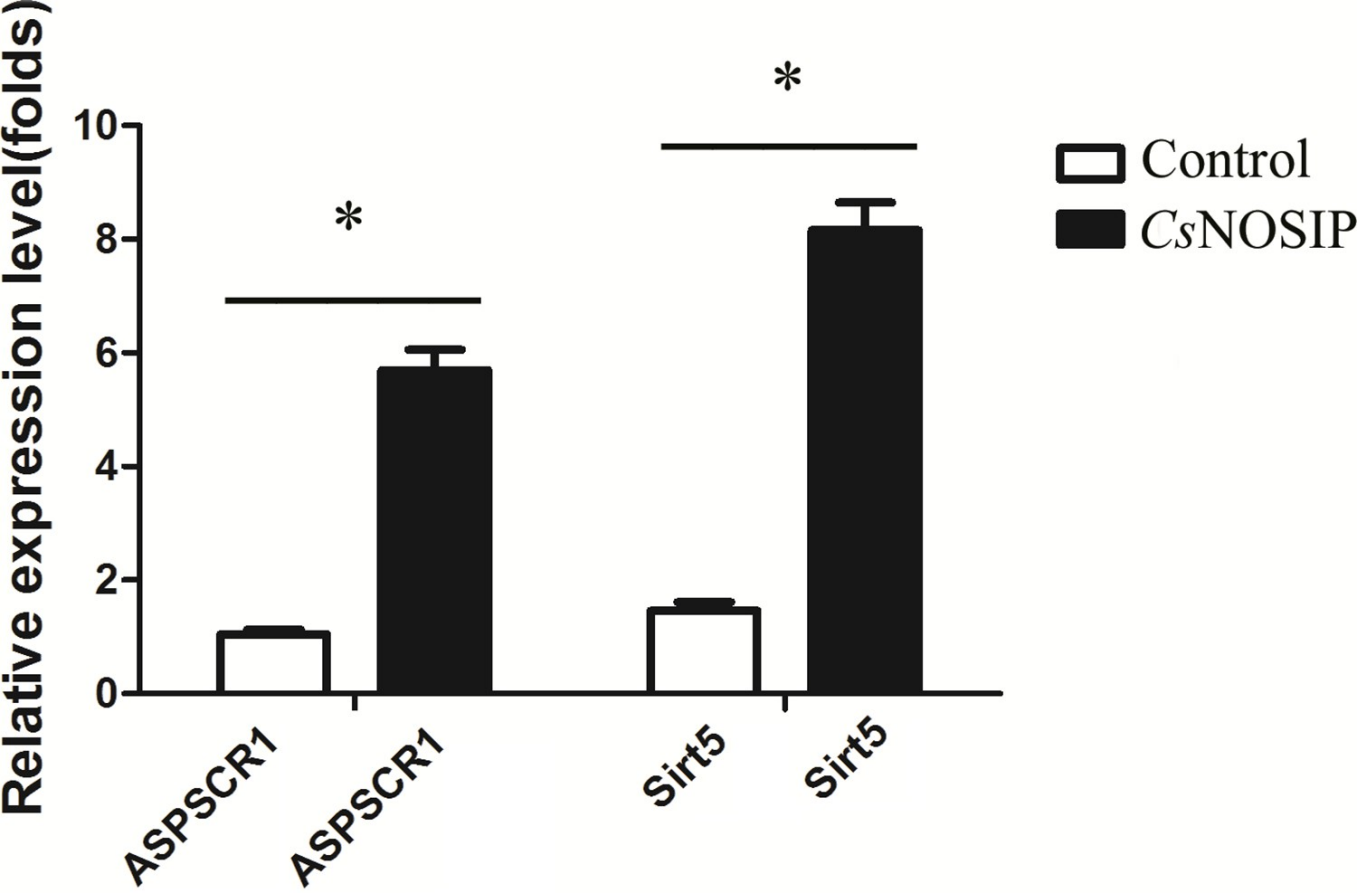
mRNA expression of ASPSCR1 and Sirt5 in CCLP-1 cells by qRT-PCR. Groups: Control: CCLP-1 cells without *Cs*NOSIP; *Cs*NOSIP: CCLP-1 cells with 5 μg/ml *Cs*NOSIP. The transcription levels of ASPSCR1 and Sirt5 are analyzed by means of the 2^−ΔΔCt^ ratio, with human β-actin employing as the transcriptional control. Data are represented as mean ± SD (*p<0.05). Cultures were set up in triplicate and data were displayed as mean ± SD. Statistical significance was analyzed by the Student’s t test (*p < 0.05).

The mRNA expression patterns of ASPSCR1 and Sirt 5 in CCLP1 cells were measured by qRT-PCR. As shown in Fig 2, compared with control group, ASPSCR1 and Sirt 5 mRNA expressions were significantly increased (*p* < 0.05).

### *Cs*NOSIP interacts with ASPSCR-1 and Sirt-5

**Fig 3:**
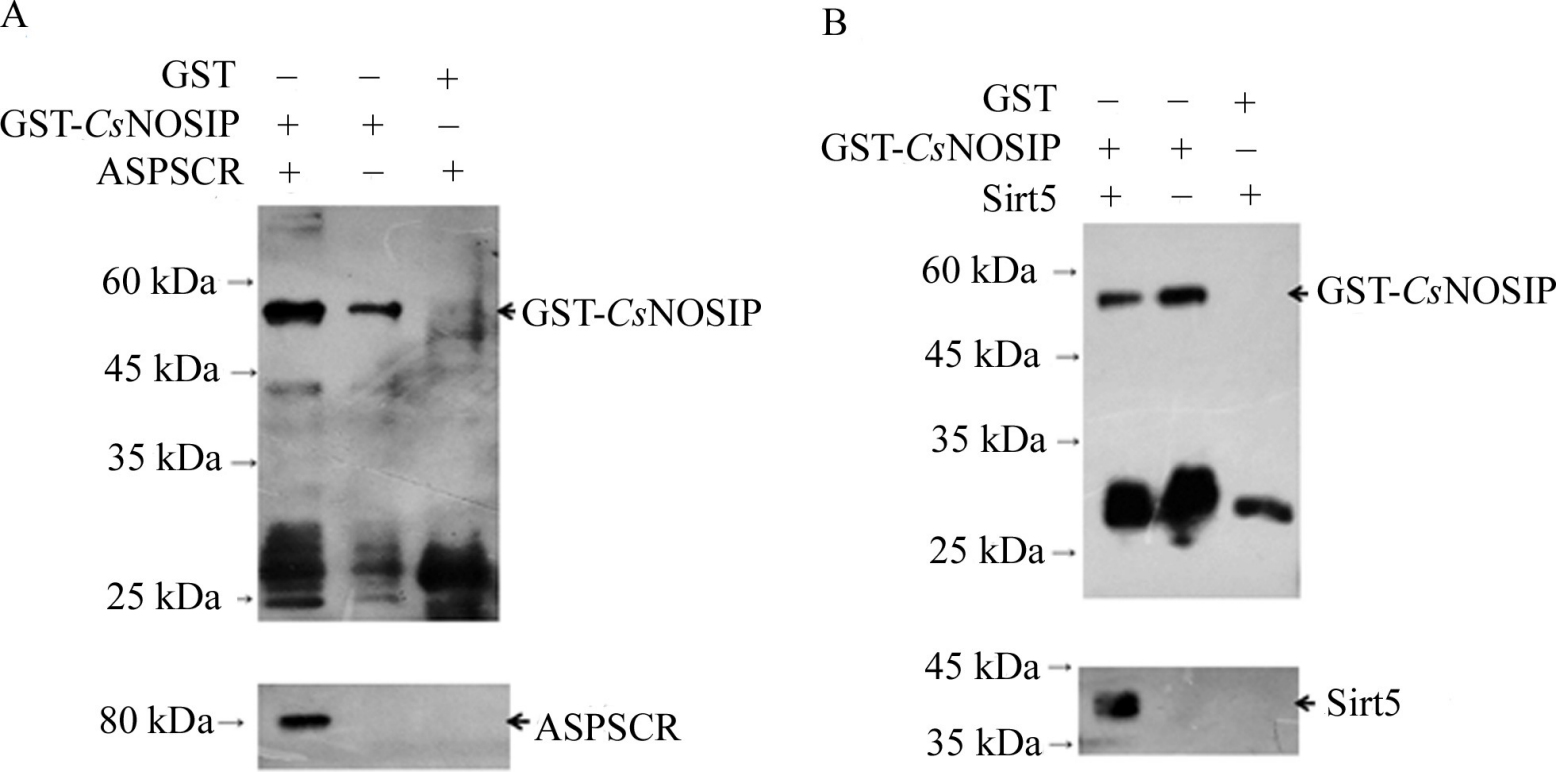
*Cs*NOSIP interacts with ASPSCR-1 and Sirt-5. Western-blot of typical protein-containing fractions collected after pull-down assay of GST-*Cs*NOSIP complexes. (A) GST-pull down assay of *Cs*NOSIP and ASPSCR-1. (B) GST-pull down assay of *Cs*NOSIP and Sirt-5.

ASPSCR-1 and Sirt-5, which may be closely related to tumor migration had been identified, were selected according to the bioinformatics analysis. Physical interaction between GST-r*Cs*NOSIP and ASPSCR-1/Sirt-5 was confirmed by GST-pull down assay. As shown in Fig 3A, in the test between GST-r*Cs*NOSIP and ASPSCR-1, the two proteins were detected simultaneously. In the test of GST-r*Cs*NOSIP without ASPSCR-1, only r*Cs*NOSIP was detected. In the pull-down test using purified GST protein and ASPSCR-1, the two proteins were not detected in the elution from GST protein coupled affinity beads. In the GST-pull down assay of r*Cs*NOSIP and Sirt-5, the same results were detected (Fig 3B). These results suggested that ASPSCR-1 and Sirt-5 interacted with r*Cs*NOSIP.

### Expression analysis of ASPSCR-1 and Sirt 5

The over-expression of ASPSCR-1/Sirt 5 was determined by western blot. As shown in S3A Fig, the expression of ASPSCR-1 and Sirt 5 were all increased by transiently transfected an over-expression vector in CCLP-1 cell lines. After 48 hours of transfection, ASPSCR-1 expression level was obvious higher than untreated cells. Compared with ASPSCR-1, over-expression of Sirt 5 needs more time (72 h) after cell transfection. Transfection of cells with specific siRNAs resulted in a large decrease in ASPSCR-1/Sirt 5 expression levels as judged by western-blot (S3B Fig) and qRT-PCR (S3C Fig). In the ASPSCR knockdown experiments, results of western-blot and qRT-PCR were consistent, ASPSCR-1 siRNAs reduced the gene expression and consequently reduced the target protein expression. Sirt 5 siRNAs showed different effects. According to the results of qRT-PCR, two Sirt 5 siRNAs significantly (*p*<0.001) reduced the target gene expression. While western-blot analysis showed that only Sirt 5 siRNA-1 restricted the expression of the target protein.

### Measurement of NO production and ROS level in CCLP-1 cells

**Fig 4:**
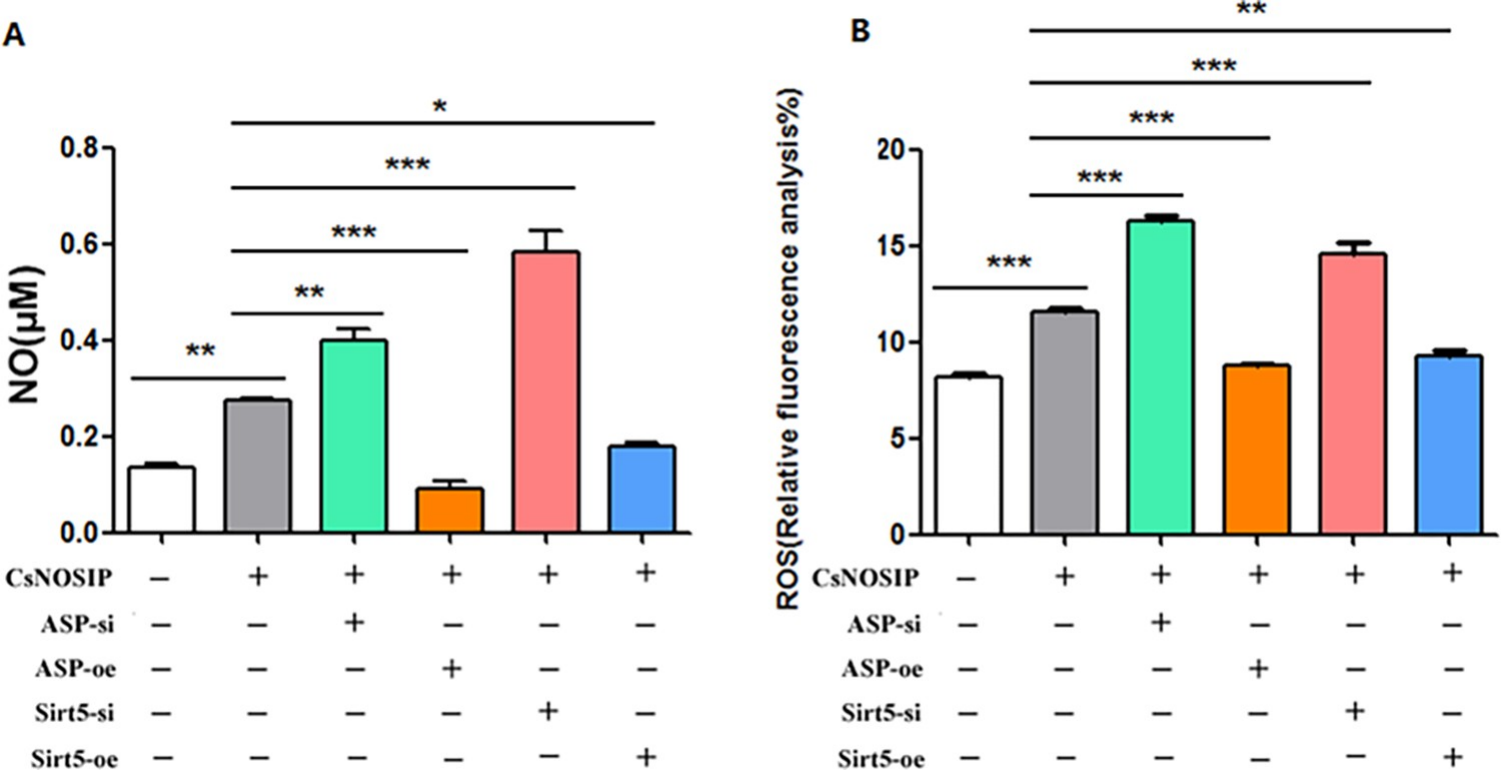
Measurement of NO production (A) and ROS (B) in CCLP-1 cells. Groups: *Cs*NOSIP-: CCLP-1 cells without stimulation; *Cs*NOSIP+: CCLP-1 cells stimulated by *Cs*NOSIP; Asp-si-*Cs*NOSIP+: ASPSCR1 siRNA inhibition CCLP-1 cells stimulated by *Cs*NOSIP; Asp-oe-*Cs*NOSIP+: ASPSCR1 over-expression CCLP-1 cells stimulated by *Cs*NOSIP; Sirt5-si-*Cs*NOSIP+: Sirt5 siRNA inhibition CCLP-1 cells stimulated by *Cs*NOSIP; Sirt5-oe-*Cs*NOSIP+: Sirt5 over-expression CCLP-1 cells stimulated by *Cs*NOSIP. Assays were performed in triplicate and data were displayed as mean ± SD. Statistical significance was analyzed by the one-way ANOVA test (*p <0.05, **p < 0.01, ***p < 0.001).

NO and ROS have been implicated in the process of CCA by inducing cell damage through the formation of toxic radicals. Hence, we tested the influence of NO by different CCLP-1 cells treated with r*Cs*NOSIP. As shown in Fig 4A, compared to control *Cs*- group, control *Cs*+ group significantly increased NO production in CCLP-1 cells (Fig 4A). Interestingly, NO production was increased inASPSCR-1/Sirt-5 low expression (ASP-si or Sirt5-si) CCLP-1 cells (Fig 4A) but decreased in Asp-oe or Sirt5-oe CCLP-1 cells (Fig 4A) when stimulated with r*Cs*NOSIP. In addition, ROS results was shown in Fig 4B, in agreement with NO results, indicated that there were significant increase in ASP-si or Sirt5-si CCLP-1 cells (Fig 4B) but decrease in ASPSCR-1/Sirt-5 over expression (Asp-oe or Sirt5-oe) CCLP-1 cells (Fig 4B) when stimulated with r*Cs*NOSIP (Fig 4B)

### *Cs*NOSIP stimulates proliferation and migration

**Fig 5:**
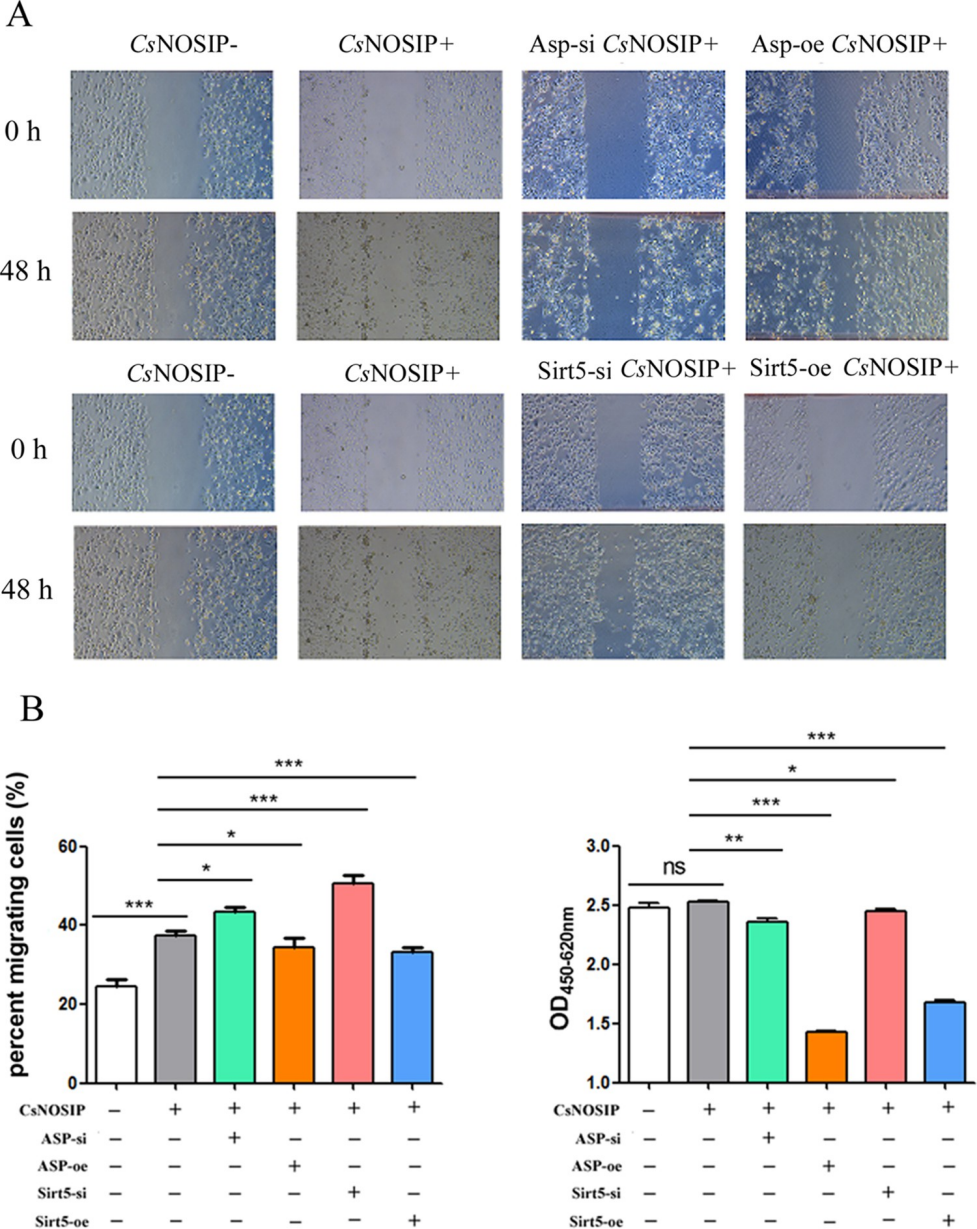
The effect of *Cs*NOSIP on CCLP1 cells. (A) Cell migration of CCLP1 cells shown by wound-healing assays; cells were observed using a light microscope under 10× objective. (B) Migration of different cells treated by *Cs*NOSIP. (C) Cell proliferation level measured by CCK-8 assay. Relative cell migration level was calculated by normalizing to cell migration level at 0 h. Groups: *Cs*NOSIP-: CCLP-1 cells without stimulation; *Cs*NOSIP+: CCLP-1 cells stimulated by *Cs*NOSIP; Asp-si- *Cs*NOSIP+: ASPSCR1 siRNA inhibition CCLP-1 cells stimulated by *Cs*NOSIP; Asp-oe-*Cs*NOSIP+: ASPSCR1 over-expression CCLP-1 cells stimulated by *Cs*NOSIP; Sirt5-si-*Cs*NOSIP+: Sirt5 siRNA inhibition CCLP-1 cells stimulated by *Cs*NOSIP; Sirt5-oe-*Cs*NOSIP+: Sirt5 over-expression CCLP-1 cells stimulated by *Cs*NOSIP. Assays were performed in triplicate and data were displayed as mean ± SD. Statistical significance was analyzed by the one-way ANOVA test (*p< 0.05, **p< 0.01, ***p< 0.001).

CCK-8 cell proliferation assay and wound healing assays were performed to investigate the effects of r*Cs*NOSIP on ASP-si/Sirt5-si or ASP-oe/Sirt5-oe CCLP1 cells. As shown in Fig 5C, after stimulation with r*Cs*NOSIP, the proliferation of CCLP1 cells slightly increased but was not changed significantly, while the migration of CCLP1 cells was increased significantly (*p*<0.001) (Fig 5B). Moreover, the proliferation and migration were increased in ASP-si or Sirt5-si CCLP-1 cells but decreased in Asp-oe or Sirt5-oe CCLP-1 cells when stimulated with r*Cs*NOSIP (Fig 5B and 5C).

## Discussion

*C*. *sinensis* infection is a major risk factor for CCA in Asian countries [7]. *C*. *sinensis*-induced CCA is closely linked to oxidative or nitrative stress for creating feasible microenvironment conducive for initiation and promotion of CCA [24]. *Cs*ESPs are capable of inducing the generation of ROS/ reactive nitrogen species (RNS) through the activation of NADPH oxidases, xanthine oxidase, and iNOS [12]. Among various components of *Cs*ESPs, r*Cs*NOSIP was identified as NOS interacting protein that upregulated the expression of iNOS in macrophage [25]. Accordingly, our results showed that ROS/NO in CCLP-1 cells increased with r*Cs*NOSIP stimulation was speculated to be resulted from the upregulation of catalytic enzymes (Fig 4). In addition to provoke oxidative or nitrative stress, *Cs*ESPs have been proven to promote proliferation and suppress apoptosis in different human cells [26,27]. Although r*Cs*NOSIP did not exhibit an obvious effect on enhancing the proliferation of CCLP-1 cells, its effect on enhancing the migration of CCLP-1 cells was similar to that of *C*. *sinensis* granulin, another ingredient of *Cs*ESPs. This indicates that *Cs*NOSIP might primarily participated in promoting CCA invasion and metastasis (Fig 5) [28].

Given the importance of *Cs*NOSIP as a key molecule in *Cs*ESPs [17,25], we identified 70 potential r*Cs*NOSIP-binding proteins (Supporting Information), which predominantly enriched in protein serine/threonine kinase activity, regulating oxidative stress-induced intrinsic apoptotic signaling pathway, and GMP-PKG signaling pathway (Fig 1B). During infection, oxidative or nitrative stress will be triggered from host cells for generating large amounts of nitrogen and oxygen radicals to eliminate pathogen. For example, NO as the production of nitrative stress can activate its downstream cGMP-dependent protein kinase G (PKG) and participate in a protective response against environmental injury [29]. Since *Cs*NOSIP can stimulate ROS/NO production (Fig 4), these r*Cs*NOSIP-binding proteins may be involved in a host defense mechanism against *C*. *sinensis* infection. Of these proteins, SIRT5 is a member of sirtuins (SIRTs) family, which are highly conserved nicotinamide adenine dinucleotide (NAD+)-dependent histone deacetylases with the ability to deacetylate histone and nonhistone targets [30,31], while no researches have reported the function of alveolar soft part sarcoma chromosomal region candidate gene 1 protein (ASPSCR-1). We firstly observed that ROS/NO production, proliferation, or migration were increased in ASP-si or Sirt5-si CCLP-1 cells but decreased in Asp-oe or Sirt5-oe CCLP-1 cells when stimulated with r*Cs*NOSIP (Figs 4 and 5). Excessive NO and other reactive oxygen intermediates will lead to fatal pathogenic consequences, such as oxidative DNA or protein damage and even cancerization [24]. Recently researches revealed a ROS elimination role of SIRT5 through activating superoxide dismutase 1 and inhibiting peroxisome [32,33]. Thus, the interaction of Sirt-5 and ASPSCR-1 with r*Cs*NOSIP implied the protective role of these two proteins in response to pathological stimulus and oxidative or nitrative damage (S3 Fig).

Collectively, our results uncovered a previously unknown function of r*Cs*NOSIP in promoting CCA invasion and metastasis as well as a novel protective role of SIRT5/ ASPSCR1 against adverse stimulus, which partly support the acceptable theory that *Cs*ESPs induce the occurrence and progression of CCA through ROS/RNS-induced oxidative and nitrative DNA damage [34]. Further investigations are required to clarify the precise mechanisms for providing therapeutic strategies of clonorchiasis.

## Supporting information

S1 Fig Ni2+-NTA chromatographyl. UV280 nm cure(blue), Programmed %B cure(green) and conductivity cure(red) were show in the graph. (TIF)

S2 Fig Identification of r*Cs*NOSIP-binding proteins. (A) The whole protein chip scanning result comprising 21,000 probes. (B) Enlarged piture of one block in this chip assay. Red arrow indicated the positive control. Blue arrow indicated the negative control. Yellow arrow indicated the exhibition spot of positive protein.
(TIF)

S3 Fig Expression analysis of ASPSCR-1 and Sirt-5 in different condition. (A) Western blot to examine over-expression ASPSCR-1/Sirt-5 in CCLP-1 cells. Lane 1: cells without transfection; Lane 2: cells transfected with an over-expression vector and cultured for 48 h; Lane 3: cell transfected with an over-expression vector and cultured for 72 h. (B) Western blot to examine expression ASPSCR-1/Sirt-5 in CCLP-1 cells. Cells were collected at 72 h after transfected with siRNA. Lane 4: ASPSCR-1/Sirt-5 expression interfered by siRNA 1 group; lane 5: ASPSCR-1/Sirt-5 expression interfered by siRNA 2 group; Lane 6: ASPSCR-1/Sirt-5 expression without siRNA. QPCR to examine expression ASPSCR-1/Sirt-5 in CCLP-1 cells. Cells were collected at 72 h after transfected with siRNA. (C) Results of ASPSCR-1 gene expression treated by siRNAs. (D) Results of Sirt-5 gene expression treated by siRNAs. Assays were performed in triplicate and data were displayed as mean ± SD. Statistical significance was analyzed by the Student’s t test (***p < 0.001).
(TIF)

S1 Table Potencial proteins interaction with *Cs*NOSIP. (DOCX)
