# Supplementary figures and images for: ASPSCR-1 and Sirt-5 alleviate Clonorchis liver fluke rCsNOSIP-induced oxidative stress, proliferation, and migration in cholangiocarcinoma cells
Source: PLoS Negl Trop Dis. 2023 Nov 10;17(11):e0011727. doi: 10.1371/journal.pntd.0011727 (PMC10664913; doi:10.1371/journal.pntd.0011727)

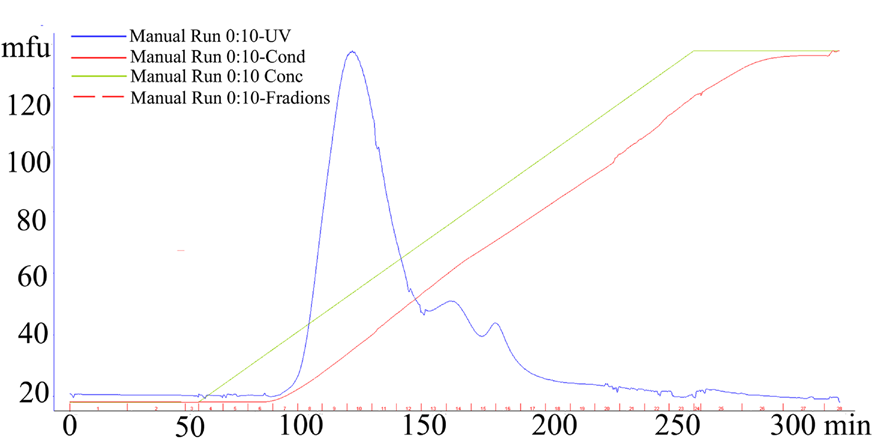

Supplement: S1 Fig — (TIF) [file pntd.0011727.s001.tif]

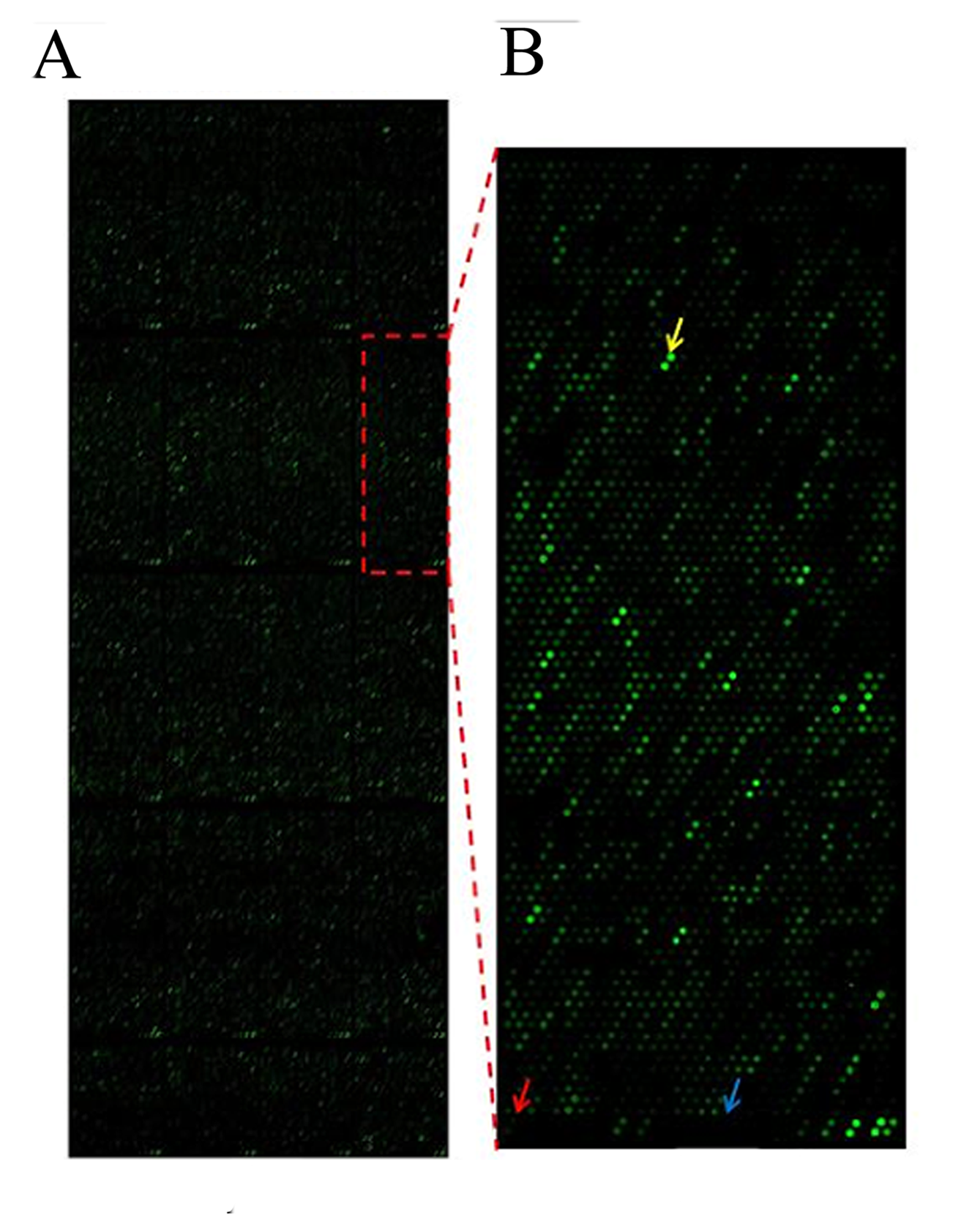

Supplement: S2 Fig — (A) The whole protein chip scanning result comprising 21,000 probes. (B) Enlarged piture of one block in this chip assay. Red arrow indicated the positive control. Blue arrow indicated the negative control. Yellow arrow indicated the exhibition spot of positive protein. (TIF) [file pntd.0011727.s002.tif]

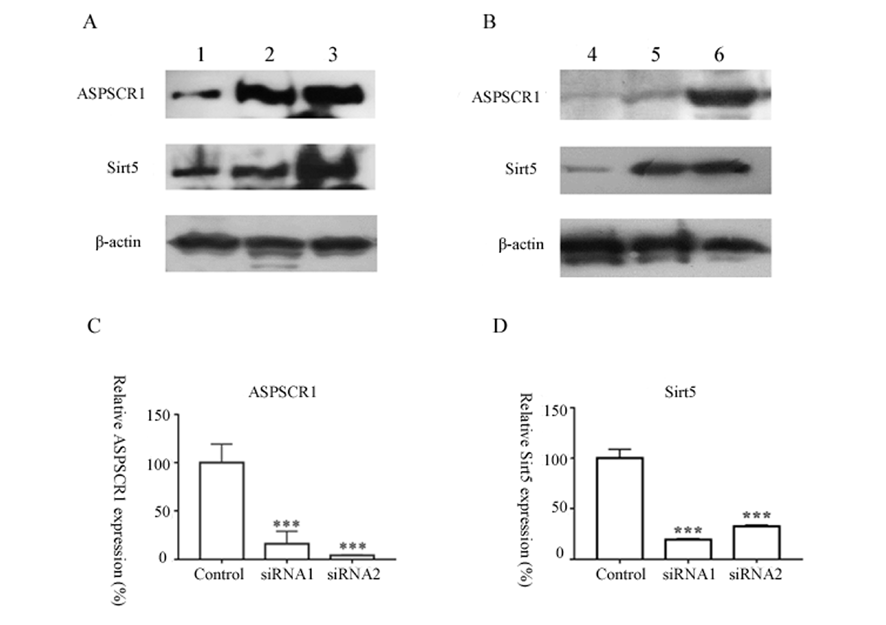

Supplement: S3 Fig — (A) Western blot to examine over-expression ASPSCR-1/Sirt-5 in CCLP-1 cells. Lane 1: cells without transfection; Lane 2: cells transfected with an over-expression vector and cultured for 48 h; Lane 3: cell transfected with an over-expression vector and cultured for 72 h. (B) Western blot to examine expression ASPSCR-1/Sirt-5 in CCLP-1 cells. Cells were collected at 72 h after transfected with siRNA. Lane 4: ASPSCR-1/Sirt-5 expression interfered by siRNA 1 group; lane 5: ASPSCR-1/Sirt-5 expression interfered by siRNA 2 group; Lane 6: ASPSCR-1/Sirt-5 expression without siRNA. QPCR to examine expression ASPSCR-1/Sirt-5 in CCLP-1 cells. Cells were collected at 72 h after transfected with siRNA. (C) Results of ASPSCR-1 gene expression treated by siRNAs. (D) Results of Sirt-5 gene expression treated by siRNAs. Assays were performed in triplicate and data were displayed as mean ± SD. Statistical significance was analyzed by the Student’s t test (***p < 0.001). (TIF) [file pntd.0011727.s003.tif]
