# Supplementary material for: ASPSCR-1 and Sirt-5 alleviate Clonorchis liver fluke rCsNOSIP-induced oxidative stress, proliferation, and migration in cholangiocarcinoma cells
Source: PLoS Negl Trop Dis. 2023 Nov 10;17(11):e0011727. doi: 10.1371/journal.pntd.0011727 (PMC10664913; doi:10.1371/journal.pntd.0011727)
Supplement: S1 Table — (DOCX) [file pntd.0011727.s004.docx]

| Block | Column | Row | Name | ID | SNR-CsNOSIP | SNR-BSA | Normalizat  ion-CsNOSIP | Normaliza  tion-BSA | FC |
| --- | --- | --- | --- | --- | --- | --- | --- | --- | --- |
| 5 | 25 | 37 | ASPSCR1 | JHU08263.B5C25R37 | 18.55 | 0.99 | 6.64 | 0.33 | 20.26 |
| 9 | 19 | 11 | DLG4 | JHU12085.B9C19R11 | 27.77 | 3.45 | 9.95 | 1.14 | 8.71 |
| 11 | 31 | 65 | DLG3 | JHU15594.B11C31R65 | 82.07 | 10.46 | 29.40 | 3.46 | 8.49 |
| 4 | 11 | 11 | MAPK1 | JHU00626.B4C11R11 | 56.93 | 14.00 | 20.40 | 4.63 | 4.40 |
| 13 | 11 | 9 | PHYHIPL | JHU09949.B13C11R9 | 16.68 | 5.18 | 5.97 | 1.71 | 3.48 |
| 13 | 27 | 75 | CCDC6 | JHU16261.B13C27R75 | 18.12 | 5.79 | 6.49 | 1.92 | 3.38 |
| 13 | 14 | 73 | SATB1 | JHU18221.B13C14R73 | 15.73 | 5.30 | 5.63 | 1.75 | 3.21 |
| 9 | 26 | 13 | POGZ | JHU12321.B9C26R13 | 23.44 | 8.01 | 8.40 | 2.65 | 3.17 |
| 7 | 18 | 71 | ACBD3 | JHU10271.B7C18R71 | 17.17 | 6.63 | 6.15 | 2.20 | 2.80 |
| 10 | 17 | 13 | GLRX5 | JHU12382.B10C17R13 | 18.74 | 7.56 | 6.71 | 2.50 | 2.68 |
| 10 | 11 | 53 | PPIA | JHU14774.B10C11R53 | 21.38 | 8.77 | 7.66 | 2.90 | 2.64 |
| 2 | 19 | 81 | EXOSC5 | JHU05214.B2C19R81 | 15.40 | 7.01 | 5.52 | 2.32 | 2.38 |
| 9 | 3 | 57 | PLCXD3 | JHU14963.B9C3R57 | 15.12 | 6.90 | 5.42 | 2.28 | 2.37 |
| 5 | 13 | 31 | AHNAK2 | JHU07968.B5C13R31 | 18.29 | 8.37 | 6.55 | 2.77 | 2.37 |
| 1 | 9 | 67 | SORBS3 | JHU04395.B1C9R67 | 19.61 | 9.24 | 7.03 | 3.06 | 2.30 |
| 4 | 12 | 11 | GAPDH | JHU00420.B4C12R11 | 96.89 | 48.24 | 34.71 | 15.97 | 2.17 |
| 4 | 28 | 39 | DCTPP1 | JHU02685.B4C28R39 | 19.39 | 10.09 | 6.95 | 3.34 | 2.08 |
| 8 | 11 | 45 | TRIM28 | JHU08723.B8C11R45 | 16.84 | 8.94 | 6.03 | 2.96 | 2.04 |
| 3 | 4 | 77 | MAPK1IP1L | JHU04809.B3C4R77 | 20.82 | 11.18 | 7.46 | 3.70 | 2.02 |
| 5 | 17 | 27 | CCDC94 | JHU07414.B5C17R27 | 16.23 | 9.00 | 5.81 | 2.98 | 1.95 |
| 12 | 3 | 67 | CRYBB3 | JHU15689.B12C3R67 | 18.77 | 10.45 | 6.72 | 3.46 | 1.94 |
| 2 | 17 | 67 | MAB21L1 | JHU04362.B2C17R67 | 15.99 | 9.04 | 5.73 | 2.99 | 1.91 |
| 6 | 7 | 49 | SF3B4 | JHU09195.B6C7R49 | 129.65 | 74.44 | 46.45 | 24.64 | 1.89 |
| 10 | 30 | 53 | LASP1 | JHU14849.B10C30R53 | 15.94 | 9.18 | 5.71 | 3.04 | 1.88 |
| 6 | 6 | 61 | SSBP3 | JHU09872.B6C6R61 | 47.47 | 28.17 | 17.01 | 9.33 | 1.82 |
| 5 | 1 | 47 | ADAT3 | JHU08691.B5C1R47 | 16.84 | 10.12 | 6.03 | 3.35 | 1.80 |
| 13 | 5 | 83 | NAT6 | JHU03423.B13C5R83 | 17.31 | 10.42 | 6.20 | 3.45 | 1.80 |
| 13 | 16 | 77 | UBXN1 | JHU00436.B13C16R77 | 17.04 | 10.31 | 6.10 | 3.41 | 1.79 |
| 12 | 19 | 89 | FRS2 | JHU13340.B12C19R89 | 17.25 | 10.59 | 6.18 | 3.51 | 1.76 |
| 13 | 32 | 65 | FKBP15 | JHU17896.B13C32R65 | 17.79 | 11.55 | 6.37 | 3.82 | 1.67 |
| 10 | 3 | 55 | SPIN1 | JHU14972.B10C3R55 | 18.15 | 11.89 | 6.50 | 3.94 | 1.65 |
| 1 | 8 | 75 | ZFYVE19 | JHU04797.B1C8R75 | 18.56 | 12.22 | 6.65 | 4.05 | 1.64 |
| 14 | 15 | 73 | ACOT7 | JHU13980.B14C15R73 | 17.08 | 11.31 | 6.12 | 3.74 | 1.63 |
| 14 | 12 | 43 | PPA1 | JHU18215.B14C12R43 | 23.25 | 15.45 | 8.33 | 5.11 | 1.63 |
| 13 | 6 | 47 | TLK1 | JHU17279.B13C6R47 | 18.00 | 11.97 | 6.45 | 3.96 | 1.63 |
| 1 | 31 | 65 | OVOL2 | JHU04191.B1C31R65 | 15.33 | 10.20 | 5.49 | 3.37 | 1.63 |
| 16 | 20 | 87 | KCTD14 | JHU05041.B16C20R87 | 16.14 | 11.08 | 5.78 | 3.67 | 1.58 |
| 6 | 24 | 27 | SORBS1 | JHU07575.B6C24R27 | 29.54 | 20.35 | 10.58 | 6.73 | 1.57 |
| 17 | 17 | 17 | GTF2I | JHU16415.B17C17R17 | 15.83 | 11.21 | 5.67 | 3.71 | 1.53 |
| 15 | 9 | 79 | KHSRP | JHU19394.B15C9R79 | 24.15 | 17.11 | 8.65 | 5.66 | 1.53 |
| 5 | 7 | 85 | KIAA0907 | JHU11460.B5C7R85 | 24.98 | 17.75 | 8.95 | 5.87 | 1.52 |
| 17 | 4 | 17 | AKT1 | JHU25302.B17C4R17 | 16.49 | 11.73 | 5.91 | 3.88 | 1.52 |
| 6 | 3 | 65 | DAB1 | JHU10095.B6C3R65 | 20.67 | 14.79 | 7.41 | 4.89 | 1.51 |
| 11 | 1 | 5 | SRRT | JHU11715.B11C1R5 | 15.35 | 11.08 | 5.50 | 3.67 | 1.50 |
| 16 | 9 | 17 | PPIE | JHU04290.B16C9R17 | 19.21 | 14.29 | 6.88 | 4.73 | 1.45 |
| 9 | 23 | 3 | TRIM24 | JHU11599.B9C23R3 | 20.45 | 15.47 | 7.33 | 5.12 | 1.43 |
| 14 | 25 | 63 | MAGI1 | JHU18003.B14C25R63 | 35.41 | 26.85 | 12.69 | 8.89 | 1.43 |
| 5 | 6 | 45 | FSIP1 | JHU07906.B5C6R45 | 27.95 | 21.21 | 10.01 | 7.02 | 1.43 |
| 6 | 30 | 89 | HAO1 | JHU11452.B6C30R89 | 20.87 | 15.90 | 7.48 | 5.26 | 1.42 |
| 1 | 10 | 41 | CRIP2 | JHU02613.B1C10R41 | 28.60 | 22.17 | 10.25 | 7.34 | 1.40 |
| 6 | 9 | 79 | UNG | JHU10923.B6C9R79 | 34.01 | 26.44 | 12.18 | 8.75 | 1.39 |
| 9 | 27 | 15 | IRF2BP1 | JHU12393.B9C27R15 | 19.18 | 14.93 | 6.87 | 4.94 | 1.39 |
| 3 | 7 | 75 | WWP2 | JHU04987.B3C7R75 | 47.98 | 37.45 | 17.19 | 12.39 | 1.39 |
| 9 | 12 | 57 | SPRR3 | JHU14973.B9C12R57 | 18.80 | 14.68 | 6.74 | 4.86 | 1.39 |
| 15 | 9 | 77 | NMD3 | JHU03424.B15C9R77 | 16.09 | 12.73 | 5.77 | 4.21 | 1.37 |
| 15 | 21 | 61 | HECW2 | JHU29114.B15C21R61 | 41.18 | 32.58 | 14.76 | 10.78 | 1.37 |
| 14 | 28 | 33 | TLK1 | JHU17279.B14C28R33 | 15.41 | 12.39 | 5.52 | 4.10 | 1.35 |
| 16 | 2 | 85 | DUSP13 | JHU04922.B16C2R85 | 15.37 | 12.36 | 5.51 | 4.09 | 1.35 |
| 7 | 24 | 23 | LIMCH1 | JHU07124.B7C24R23 | 18.03 | 14.51 | 6.46 | 4.80 | 1.35 |
| 3 | 28 | 73 | GIMAP4 | JHU04931.B3C28R73 | 48.62 | 39.42 | 17.42 | 13.05 | 1.33 |
| 6 | 3 | 47 | QKI | JHU08508.B6C3R47 | 19.91 | 16.70 | 7.13 | 5.53 | 1.29 |
| 3 | 10 | 75 | NFYC | JHU04956.B3C10R75 | 50.09 | 42.03 | 17.95 | 13.91 | 1.29 |
| 16 | 31 | 79 | ZFAND2B | JHU03645.B16C31R79 | 17.31 | 14.53 | 6.20 | 4.81 | 1.29 |
| 1 | 31 | 81 | GPKOW | JHU05314.B1C31R81 | 29.90 | 25.14 | 10.71 | 8.32 | 1.29 |
| 5 | 15 | 71 | PICALM | JHU10045.B5C15R71 | 16.44 | 13.99 | 5.89 | 4.63 | 1.27 |
| 12 | 5 | 47 | SRXN1 | JHU14249.B12C5R47 | 21.17 | 18.03 | 7.58 | 5.97 | 1.27 |
| 9 | 9 | 3 | HOMER2_frag | JHU11649.B9C9R3 | 17.85 | 15.43 | 6.39 | 5.11 | 1.25 |
| 13 | 6 | 77 | UBXN1 | JHU00329.B13C6R77 | 16.52 | 14.57 | 5.92 | 4.82 | 1.23 |
| 9 | 3 | 29 | CBLN4 | JHU13030.B9C3R29 | 16.82 | 15.02 | 6.03 | 4.97 | 1.21 |
| 1 | 14 | 53 | PRRC2B | JHU03219.B1C14R53 | 125.29 | 112.60 | 44.89 | 37.27 | 1.20 |
